# Fasting, Systemic Epigallocatechin-3-Gallate Exposure, and Hepatotoxicity

**DOI:** 10.3390/ijms27219420

**Published:** 2026-10-22

**Authors:** Ayşegül Sümer

**Affiliations:** Department of Medical Biochemistry, Faculty of Medicine, Recep Tayyip Erdoğan University, 53100 Rize, Türkiye; aysegul.sumer@erdogan.edu.tr

**Keywords:** green tea, epigallocatechin-3-gallate, EGCG, fasting, systemic exposure, hepatotoxicity, green tea extract, drug-induced liver injury, evidence appraisal

## Abstract

Green tea (*Camellia sinensis*) contains bioactive catechins, particularly epigallocatechin-3-gallate (EGCG), but traditional infusions and concentrated green tea extracts differ in EGCG content, dosing pattern, product matrix, systemic exposure, and safety. This structured narrative review evaluates how fasting affects EGCG pharmacokinetics and the relevance of increased systemic exposure to hepatotoxicity. Its distinctive contribution is not a further summary of green tea safety but an explicit appraisal of the evidence chain that is generally assumed to connect the two: each link is assessed separately, and the steps that are demonstrated in humans are separated from those that rest on biological inference. Standardized catechin preparations or purified EGCG taken after an overnight fast produce substantially higher peak plasma concentrations and total systemic exposure than when taken with food, although the two randomized within-subject comparisons that establish this effect were small, differed in dose and formulation, and assessed neither hepatic exposure nor liver injury. Evidence linking high EGCG exposure to prooxidant effects, mitochondrial dysfunction, and hepatocellular injury derives mainly from cellular and animal models at concentrations that exceed reported human peak plasma concentrations by roughly one order of magnitude; in mice, caloric restriction renders otherwise non-hepatotoxic EGCG doses hepatotoxic. In humans these outcomes must be kept apart. High-dose, long-term extract use raises serum aminotransferases in a susceptible minority, a laboratory abnormality demonstrated in controlled trials; hepatic adverse events cluster among catechin-rich bolus preparations rather than brewed beverages; and clinically apparent liver injury, documented in registry cases and associated with HLA-B*35:01, is idiosyncratic rather than dose-determined. Progression from the laboratory abnormality to clinically apparent injury has not been established. Fasting therefore increases systemic EGCG exposure, particularly from concentrated preparations, but has not been established as an independent cause of hepatotoxicity in humans. Treating fasting as an exposure-modifying condition rather than a hepatotoxic factor and nutritional state as a graded variable rather than a single category clarifies which precautions are evidence-based and which research questions remain genuinely open. Safety assessments should consider product type, dose, bolus administration, fed or fasting state, concomitant exposures, and individual susceptibility.

## 1. Introduction

Green tea (*Camellia sinensis*) is produced by limiting the oxidation of polyphenolic compounds during leaf processing, thereby preserving substantial amounts of monomeric catechins [1]. The principal catechins in green tea are epigallocatechin-3-gallate (EGCG), epigallocatechin (EGC), epicatechin-3-gallate (ECG), and epicatechin (EC), with EGCG being the most abundant in commercially available green tea products [2]. Because the oral bioavailability of EGCG is low and variable, the assessment of its biological effects should consider systemic exposure rather than the ingested amount alone [3,4].

Because green tea is consumed in different product forms, traditionally prepared infusions and concentrated green tea extracts should not be regarded as equivalent exposures. Traditional infusions are generally considered safe at the reported consumption levels, whereas concentrated extracts may deliver higher and more variable amounts of EGCG; their composition, manufacturing process, and mode of use can influence systemic exposure [1,5]. The fed or fasting status is an important pharmacokinetic variable: administration of a standardized green tea catechin preparation after an overnight fast markedly increases systemic exposure to unconjugated EGCG compared with administration with a light meal [6]. The United States Pharmacopeia (USP) assessment also identifies fasting and bolus dosing as conditions that may increase EGCG bioavailability [5].

Much of the mechanistic evidence linking high EGCG exposure to oxidative stress, mitochondrial dysfunction, and hepatocellular injury has been derived from cellular and animal models [7,8]. In humans, controlled clinical data indicate that prolonged use of high-dose green tea extract can increase serum aminotransferases in some individuals, whereas a hepatocellular pattern and marked interindividual susceptibility characterize clinically apparent green tea-associated liver injury [9,10]. Nevertheless, no controlled causal study has shown that the increased bioavailability observed under fasting conditions directly translates to a higher incidence of clinical hepatotoxicity in humans [1,5].

The hepatic safety of green tea catechins has been reviewed repeatedly, and two assessments are particularly authoritative: the European Food Safety Authority (EFSA) scientific opinion on green tea catechins [1] and the USP comprehensive review of green tea extract hepatotoxicity [5], the latter extending an earlier USP systematic review [11]. Independent systematic reviews have evaluated adverse events across human intervention studies [12,13] and derived exposure limits for beverages and supplements [14]. The present review does not duplicate those efforts. Each of them answers the question of what level of intake can be regarded as safe; none takes as its primary object the structure of the evidence that is assumed to link fasting-dependent exposure to clinical liver injury.

This review examines the changes in systemic EGCG exposure under fasting conditions across different forms of green tea consumption and integrates evidence on EGCG bioavailability and metabolism, oxidative and mitochondrial toxicity mechanisms, experimental hepatotoxicity, controlled human studies, clinical liver injury, and regulatory safety assessments. Its central aim is to distinguish the directly demonstrated steps in the evidence chain linking pharmacokinetic exposure to clinical hepatotoxicity from relationships that remain inferential.

### Novelty and Contribution of This Review

The contribution of this review is an integrated exposure-to-toxicity appraisal in which each link of the presumed causal chain is evaluated separately and by its own standard of evidence. Five specific contributions follow from this approach. First, the review assembles fasting-dependent pharmacokinetics, mechanistic hepatotoxicity, controlled human safety data, clinical liver injury registries, and regulatory evaluation into a single framework, and then asks explicitly which transitions between these levels are supported by direct observation. Second, it separates demonstrated increases in systemic EGCG exposure from demonstrated clinical hepatotoxicity, and reports the approximate quantitative margin between human peak plasma concentrations and the concentrations at which injury is observed experimentally, a comparison that is rarely made explicit. Third, it treats nutritional state as a graded exposure-modifying variable rather than a single category, distinguishing an overnight fast in healthy humans from sustained caloric restriction in animals and from intentional energy restriction during weight loss. Fourth, it integrates exposure with susceptibility, considering dose, formulation, bolus administration, nutritional state, mitochondrial effects, and immunogenetic predisposition as interacting rather than alternative determinants of outcome. Fifth, it identifies where biological plausibility is well supported but direct human causal evidence is absent and converts those gaps into specific research questions. The intended result is not a new safety threshold but a clearer account of what the existing evidence does and does not establish, which is a prerequisite for both proportionate precaution and well-targeted future study.

## 2. Literature Search Strategy

This study was designed as a structured narrative review and is reported in accordance with the principles of transparent narrative synthesis; it is not a systematic review and no PRISMA workflow or meta-analysis was undertaken. The primary bibliographic source was PubMed/MEDLINE, searched from database inception to 19 October 2026. Two Boolean queries were used, with all terms searched in the title and abstract fields and truncation denoted by an asterisk. The first addressed food-effect pharmacokinetics: (“epigallocatechin gallate” OR epigallocatechin OR EGCG OR “green tea”) AND (fasting OR fasted OR “fed state” OR “food effect” OR food OR meal OR postprandial) AND (bioavailability OR “systemic exposure” OR pharmacokinet* OR absorption). The second addressed hepatic safety: (“epigallocatechin gallate” OR epigallocatechin OR EGCG OR “green tea”) AND (hepatotoxic* OR “liver injury” OR “liver damage” OR DILI OR aminotransferase*). No language restriction was applied at the search stage; every study that met the eligibility criteria was available in English. No publication-year limit was applied; the predominance of references from 2005 to 2025 reflects the period in which the relevant primary studies were published rather than a restriction imposed on the search. Additional studies were identified through backward reference searching of retrieved articles and of the EFSA and USP assessments, and bibliographic details of the cited literature were checked against Crossref records.

Titles and abstracts were screened against the eligibility criteria summarised in Table 1, and the full texts of potentially eligible records were examined. Screening and selection were performed by a single author; duplicate independent screening was not possible, so no formal measure of selection reliability is available. Because the review draws on a single bibliographic database, records indexed only in Embase, Scopus, Web of Science or the CENTRAL trials register may not have been retrieved, a risk that is greatest for small pharmacokinetic studies and for conference material. The search was therefore repeated on the date given above, with particular attention to human food-effect pharmacokinetics and to hepatic safety. That updated search identified two studies now incorporated here: the randomized crossover study of Andreu-Fernández et al. [15], discussed in Section 6 and included in the corresponding summary tables, and the randomized crossover study of Zhang et al. [16], discussed at the end of Section 6. No further eligible human fed-versus-fasting comparison was identified. The human pharmacokinetic evidence base assembled here should therefore be regarded as the most complete the author could identify rather than as the product of an exhaustive multi-database search.

Regulatory and pharmacopoeial documents were not retrieved through the bibliographic search. They were identified by direct consultation of the publishing bodies’ own websites—EFSA, the European Commission (EUR-Lex), the USP, the UK Committee on Toxicity of Chemicals in Food, Consumer Products and the Environment (COT), and Health Canada—searched in October 2026 using the terms “green tea,” “green tea extract,” “catechins,” and “epigallocatechin gallate.” Only documents available in full text from the issuing body were used, and each is cited to the specific document consulted rather than to an institutional home page. Where a document has been superseded or revised, the version consulted and its date are given in the reference list.

Priority was given to human pharmacokinetic studies directly comparing fed and fasting conditions, controlled clinical trials, experimental hepatotoxicity studies, DILI registries incorporating formal causality assessments, and regulatory risk evaluations. Findings reported only in secondary sources, studies without adequate product characterization, and evidence in which EGCG or green tea exposure could not be distinguished were not used to establish principal conclusions. Cases involving multi-ingredient preparations were considered with explicit acknowledgment of the limitations of causality and product verification.

Evidence was synthesized at five levels: human pharmacokinetics, cellular and animal toxicology, controlled human safety data, clinical DILI registries, and regulatory evaluation. The study design, product and dose characteristics, definition of fasting, outcomes, and principal limitations were compared. This approach is not a systematic review or meta-analysis; rather, it aims to separate directly demonstrated associations from biologically plausible but still inferential steps.

## 3. Definition, Consumption Forms, and Catechin Profile of Green Tea

Green tea is derived from *Camellia sinensis* leaves and differs from black and oolong teas in its catechin profile because oxidation of polyphenols is limited during production [1]. Catechins account for a substantial proportion of green tea polyphenols, with EGCG, EGC, ECG, and EC among the principal compounds [2]. Chemical composition varies with cultivar, growing conditions, leaf characteristics, processing methods, and preparation of the final product [1,2].

Green tea is consumed in several forms. Traditionally, loose leaves or tea bags are infused in hot water, whereas matcha is prepared by dispersing finely ground green tea powder in hot water [1]. Other products include reconstituted beverages made from dried extracts and more concentrated liquid or dry extracts, particularly those used in dietary supplements [5]. A shared botanical origin does not imply equivalent catechin composition or systemic EGCG exposure [1]. In a comparison of fifteen commercial products, EGCG concentrations ranged from 23.3 to 70.2 mg/g across bagged, gunpowder, and matcha green teas, and catechin profiles clustered mainly by product form rather than by botanical identity alone [2].

The EGCG content of traditional infusions is not constant. Leaf quantity and characteristics, water temperature and composition, brewing duration [17], and storage conditions affect catechin extraction; consequently, cup number alone cannot define a fixed EGCG dose across products and consumers [1]. Brewing temperature and time act jointly: catechin yield and the relative proportions of individual catechins change with both, and prolonged brewing at high temperature promotes epimerization of EGCG [17]. Epimerization and degradation are not artifacts of brewing alone: EGCG forms epimers and dimers under conditions commonly used in laboratory work [18], and its stability in liquid formulations is a recognised problem in its own right [19]. In extract preparations, extraction, concentration, and manufacturing processes can substantially alter the catechin profile, making verified EGCG content important for safety assessment [1,5].

EFSA estimated mean adult EGCG exposure from traditional green tea infusions at approximately 90–300 mg/day and exposure in high consumers at up to 866 mg/day. Traditionally prepared infusions were considered generally safe at reported intake levels, although rare idiosyncratic liver injury has been reported [1]. By contrast, catechin profiles and patterns of use for concentrated extracts vary markedly with manufacturing, while bolus dosing and fasting can increase systemic EGCG exposure. Traditional infusions, matcha, reconstituted tea beverages, and concentrated extracts should therefore not automatically be assigned to the same hepatic safety category [1,5].

## 4. EGCG Bioavailability and Metabolism

The oral bioavailability of EGCG is relatively low. Gastrointestinal stability, intestinal permeability, transporter-mediated efflux, and intestinal and hepatic metabolism influence the amount reaching the circulation [3,20]. Chemical instability contributes at the earliest stage, since EGCG is labile in aqueous media, so the quantity available for absorption is not fixed by the ingested dose [18,19]. Following absorption, green tea catechins undergo methylation, glucuronidation, and sulfation [21]; most ingested catechins are not absorbed in the small intestine and reach the colon, where the gut microbiota converts them into lower-molecular-weight metabolites such as phenyl-γ-valerolactones and phenolic acids [22]. Human studies of the absorption, distribution, metabolism and excretion of tea polyphenols, and broader reviews of dietary (poly)phenol bioavailability, describe this limited systemic availability and the metabolite profile it produces [4,23].

Specific enzymes and transporters account for much of this presystemic loss. Catechol-O-methyltransferase methylates EGCG, and EGCG itself inhibits the enzyme, so methylation capacity is both a determinant and a target of catechin exposure [24]. Conjugation by UDP-glucuronosyltransferases and sulfotransferases, together with efflux of catechins and their conjugates back into the intestinal lumen, limits net absorption, and the resulting first-pass metabolism differs between individual catechins [20,21]. These mechanisms are relevant to the fasting question because any condition that alters the balance between absorption and presystemic elimination will alter systemic exposure without changing the ingested dose.

The circulating metabolic profiles of the major catechins differ. Human pharmacokinetic data indicate that EGCG and ECG occur predominantly in unconjugated form in plasma, whereas larger proportions of EGC and EC circulate as glucuronide and sulfate conjugates [6]. Thus, total catechin intake alone cannot fully explain circulating parent-compound and metabolite concentrations, and metabolic transformation must be considered when evaluating internal exposure [3,4].

Direct human data on tissue distribution are much more limited than plasma pharmacokinetic data; therefore, an increase in plasma EGCG should not be assumed to indicate a proportional increase in hepatic tissue concentration [1]. This caveat is more than formal. EGCG absorbed from the intestine reaches the liver through the portal circulation before distribution to peripheral blood, so hepatic exposure may exceed peripheral plasma concentrations, and the extent of this difference in humans is not established [1]. The presence of unchanged catechins, conjugated metabolites, and microbiota-derived catabolites in the circulation also limits interpretation of the biological effects of EGCG and other catechins solely from the plasma concentration of the parent compound [22]. Interindividual differences in gut microbiota composition contribute to variability in these catabolite profiles [22].

## 5. Pharmacokinetic Framework of the Fasting Condition

Nutritional state is not a single exposure condition, and the evidence discussed in this review concerns three distinguishable states. The first is an overnight fast of 8 to 12 h followed by administration without food, which is the condition used in human EGCG pharmacokinetic studies [6,25]. The second is sustained caloric restriction, in which energy intake is reduced over days or longer; this is the condition under which EGCG hepatotoxicity has been enhanced in mice [26]. The third is intentional energy restriction during weight loss in people, which is a common context for concentrated extract use and which resembles the second condition more than the first. These states differ in duration, metabolic consequences, and the evidence available for each, and conclusions drawn under one should not be transferred to another without justification.

In this review, “fasting” therefore refers not to prolonged starvation physiology but to administration without food after the overnight fast used in EGCG pharmacokinetic studies. In healthy participants, single-dose Polyphenon E after an overnight fast was compared within individuals with administration alongside a light breakfast, demonstrating that fed status can markedly alter EGCG pharmacokinetics [6]. EFSA likewise considered fasting and bolus dosing among the conditions that influence systemic EGCG exposure [1].

Consequently, changes in glycogenolysis, ketogenesis, or energy-sensing pathways associated with prolonged starvation should not be transferred directly as evidence of the mechanism of hepatotoxicity from fasting EGCG use. The principal direct finding in humans is that fed status alters plasma EGCG pharmacokinetics [6].

Animal data indicate that host nutritional state is not pharmacokinetically or toxicologically inert: in mice, sustained caloric restriction converts EGCG doses that are harmless under normal feeding into hepatotoxic ones [26]. That experiment is reported in full, with the limits on extrapolating it to people, in Section 8.1. What matters at this point is only which of the three states above it belongs to. The exposure condition is the second, sustained caloric restriction, and not the single overnight fast used in the human pharmacokinetic studies; the finding therefore shows that nutritional state can modify susceptibility in an animal model and does not show that an overnight fast creates toxicological susceptibility in human hepatocytes [5,26].

## 6. Effects of Fasting and Fed Status on EGCG Pharmacokinetics

In a randomized crossover study of healthy participants, Polyphenon E containing 400, 800, or 1200 mg EGCG administered after an overnight fast increased the mean maximum plasma concentration of unconjugated EGCG by more than 3.5-fold compared with administration with a light breakfast; AUC was significantly higher at all three doses [6]. The EFSA assessment of human pharmacokinetic data similarly supports that fasting and bolus administration of green tea extract can increase systemic EGCG exposure compared with fed or divided dosing [1]. For many years this was the only randomized within-subject comparison of fed and fasting administration of a standardized catechin preparation in humans, and both the EFSA and USP assessments rest on re-evaluation of these data rather than on independent trials [1,5]. A second randomized crossover study has since addressed the same question with contemporary analytical methods [15]; the two are described in turn below and compared in Table 2.

Andreu-Fernández et al. subsequently conducted a randomized three-condition crossover study in ten healthy volunteers (five men and five women, aged 25–35 years), with a seven-day washout between conditions and an overnight fast of at least eight hours before each administration [15]. Two conditions are directly relevant here: Teavigo, a purified green tea extract standardized to 94% EGCG, is given as two capsules providing 300 mg of extract with water alone, and the same preparation is given with a standardized 480-kcal breakfast. A third condition used a different product (FontUp, ≥250 mg EGCG) dissolved in milk. Plasma EGCG was quantified over six hours by UPLC-ESI-MS/MS. Mean Cmax was 528.6 ng/mL in men and 466.1 ng/mL in women under fasting conditions, compared with 275.3 and 318.7 ng/mL, respectively, with breakfast, and the corresponding AUC0–360 values were likewise numerically higher under fasting [15].

Two features of that study limit how much weight the comparison can carry. First, no exact significance level is reported for the direct fasting-versus-breakfast contrast of Cmax or AUC; the significant comparisons the authors report involve the milk-based FontUp product (for AUC0–360 against fasting Teavigo, *p* = 0.036 in men and *p* = 0.001 in women). The fasting-versus-fed difference in that study is therefore directionally consistent with Chow et al. but not independently established as statistically significant. Second, the report states the EGCG content of the Teavigo condition inconsistently, giving both a per-capsule extract mass implying approximately 282 mg EGCG and a stated total of 140 mg; the administered EGCG dose is accordingly uncertain within roughly a two-fold range. With ten participants, a six-hour sampling window, sex-stratified reporting, and no hepatic endpoint, the study enlarges the human food-effect evidence base without resolving the question of hepatic risk [15].

Taken together, the two randomized crossover studies agree in direction: administration without food raises systemic exposure to unconjugated EGCG relative to administration with food [6,15]. They differ in dose, formulation, analytical platform, sampling duration, and the comparator meal, so their effect sizes should not be pooled or treated as replicates in the strict sense. Neither measured hepatic exposure nor assessed liver injury, so the food effect remains a pharmacokinetic finding that has not been connected to a clinical hepatic outcome [1,5].

The mechanism underlying this increase has not been established. Saturation of presystemic elimination at high bolus exposure has been proposed, but it has not been shown to be the sole or definitive mechanism in humans [5]. Such saturation is biologically plausible given that methylation, glucuronidation, sulfation, and efflux all contribute to first-pass elimination and are capacity-limited [20,21,24]. Interactions of green tea catechins with food components, including dietary proteins, may also influence the amount of unconjugated EGCG available for absorption and its bioavailability [1]. Because EGCG is chemically labile in aqueous media, a food matrix may additionally alter gastric emptying and luminal conditions in ways that change how much intact EGCG reaches the absorptive epithelium [19]. These candidate mechanisms are not mutually exclusive, and no human study has yet distinguished between them.

This finding was supported by a small crossover study of purified EGCG. Four healthy participants received 500 mg EGCG after an overnight fast of at least 10 h as capsules with water alone, capsules with a light breakfast, or incorporated into strawberry sorbet. AUC0-8 after the fasting capsule was 2.7-fold higher than with breakfast and 3.9-fold higher than with sorbet; Cmax values were 824.2, 231.8, and 218.0 ng/mL, respectively [25]. Although these findings support an inhibitory effect of food on systemic absorption of both extract-associated and purified EGCG, the sample of four participants and absence of hepatic safety outcomes limit generalizability. The comparison between capsules with breakfast and EGCG incorporated into sorbet also indicates that the physical form of co-administered food, and not only its presence, affects exposure.

The relevance of these dosing conditions extends beyond pharmacokinetics. A systematic review of toxicological and human intervention data concluded that hepatotoxicity in laboratory studies was strongly associated with specific dosing conditions—in particular bolus administration by gavage and the fasted state—and was positively correlated with the total catechin and EGCG content of the preparation [12]. Experimental work supports the importance of the route and pattern of administration: in rats, repeated dosing with pure EGCG produced hepatotoxicity whose severity depended on both dose and route of administration [27]. That systematic review nevertheless assessed laboratory dosing conditions rather than a controlled fed-versus-fasting comparison of clinical outcomes in people.

A further randomized crossover study, published while this review was in revision, bears on the same question from a different direction. Zhang et al. compared a micellar green tea extract formulation co-delivered with N-acetylcysteine against a standard extract at a matched dose in 16 healthy adults and reported a 4.2-fold higher baseline-adjusted AUC0–24 and a 3.0-fold higher baseline-adjusted Cmax for EGCG with the micellar formulation (*p* = 0.032 and *p* = 0.037) [16]. All doses in that study were taken with a standardized breakfast, so it is not a fed-versus-fasting comparison and does not bear directly on the central question of Section 6. Its relevance is that formulation alone, at an identical nominal extract dose, altered systemic EGCG exposure by a factor comparable to the food effect described above. Nominal dose is therefore a poor proxy for internal exposure in either direction, which reinforces the product-form distinction drawn throughout this review [1,12]. The accompanying 30-day open-label phase in 13 participants found no significant change in liver biochemistry, but with no control group, a small sample, and a short duration, it cannot establish hepatic safety, and the study reports extract rather than EGCG doses, which limits comparison with the fed-versus-fasting literature [16].

The primary outcomes of human studies comparing fasting and fed conditions were plasma pharmacokinetic measures; hepatic EGCG concentration, oxidative stress in hepatocytes, and clinical liver injury were not evaluated [6,25]. Higher Cmax and AUC under fasting conditions therefore directly demonstrate greater systemic EGCG exposure but do not, by themselves, establish a higher incidence of hepatotoxicity in humans [5].

## 7. Mechanistic Basis of EGCG-Associated Hepatotoxicity

### 7.1. Oxidative Stress and Prooxidant Effects

Oxidative stress reflects disruption of the balance between oxidant production and antioxidant defenses sufficient to cause oxidative changes in cellular structures. Superoxide dismutase, catalase, glutathione peroxidase, and glutathione act together to maintain redox homeostasis [28]. Reactive oxygen species are not solely mediators of cellular injury: hydrogen peroxide participates in redox signaling at low physiological concentrations but can contribute to biomolecular damage at supraphysiological levels [29]. Current expert recommendations emphasize that “reactive oxygen species” is not a single functional entity and that hydrogen peroxide and superoxide should be distinguished when signaling and damage are attributed [29].

The redox properties of EGCG vary with concentration and experimental conditions. Its phenolic structure contributes to antioxidant activity, whereas EGCG auto-oxidation, particularly in cell culture, can generate superoxide and hydrogen peroxide and produce prooxidant behavior [30]. Autoxidation of EGCG also results in covalent modification of proteins, which links its prooxidant chemistry to adduct formation [31]. In primary rat hepatocytes, ROS production decreased at low EGCG concentrations but increased at the highest concentration tested, illustrating this biphasic redox behavior [7].

An important methodological caveat applies to this entire literature. EGCG added to commonly used cell culture media generates hydrogen peroxide rapidly in the absence of cells, because bicarbonate-buffered media at atmospheric oxygen tension promote auto-oxidation [32]. Some prooxidant effects attributed to EGCG in vitro may therefore reflect extracellular peroxide formation in the medium rather than an intracellular action of the parent compound, and the effect was observed across several commonly used culture media [32]. This does not invalidate in vitro findings, but it constrains their extrapolation to the hepatic milieu, where oxygen tension, bicarbonate concentration, and antioxidant capacity differ.

At sublethal exposures the same chemistry activates adaptive antioxidant responses. EGCG induces antioxidant and detoxifying enzymes through the Nrf2 pathway, which accounts for much of its cytoprotective activity at low concentrations [33]. In mice given hepatotoxic oral doses, Nrf2-dependent responses were engaged alongside changes in major antioxidant enzymes, indicating that injury occurs when oxidative burden exceeds this adaptive capacity rather than in its absence [34]. A graded transition from adaptive induction to injury is consistent with the biphasic concentration-response observed in isolated hepatocytes [7,35].

### 7.2. Mitochondrial Dysfunction

Because of their high metabolic requirements and mitochondrial density, hepatocytes are susceptible to mitochondrial toxicity, which can contribute to hepatotoxicity from drugs and xenobiotics [36]. In primary rat hepatocytes exposed to EGCG for 24 h, partial loss of mitochondrial membrane potential was observed at 30 µmol/L and near-complete loss at 100 µmol/L. In separate permeabilized-hepatocyte experiments, high EGCG concentrations were associated with uncoupling of oxidative phosphorylation and disruption of outer mitochondrial membrane integrity [7].

Mitochondrial dysfunction may impair cellular energy production and contribute to oxidative stress and cell-death pathways; recognized mechanisms include inhibition of oxidative phosphorylation, opening of the mitochondrial permeability transition pore, impaired fatty acid β-oxidation, and mitochondrial DNA damage [36]. Comparable changes have been observed in vivo. In C57BL/6J mice, intragastric EGCG at 500–750 mg/kg once daily for three days caused hepatic inflammation, necrosis, and haemorrhage; the injury was accompanied by increased oxidative stress, reduced superoxide dismutase and glutathione peroxidase, decreased hepatic mitochondrial copy number on real-time PCR and transmission electron microscopy, and lower sirtuin 3 expression [8]. Earlier work in mice had established that high oral doses of EGCG are hepatotoxic, defining the oral dose range in which hepatic injury occurs in this species [37]. Studies in isolated hepatocytes and in rodents similarly identified catechins and their oxidation products as cytotoxic at high concentrations [38]. These findings indicate that mitochondria are a plausible in vivo target of bolus EGCG rather than only an in vitro one, although the doses used greatly exceed human intake.

### 7.3. Hepatocellular Injury and Cell Death

In primary rat hepatocytes exposed to EGCG for 24 h, signs of cellular injury and reduced hepatocyte function appeared at concentrations of 10 µmol/L or higher, with mitochondrial and apoptotic changes becoming more pronounced at higher concentrations [7]. The ability of EGCG to act as a prooxidant at high concentrations and the association between ROS formation and cellular injury support oxidative stress as one potential toxicity mechanism [30]. In mouse models, injury has been characterized by hepatocellular necrosis with accompanying apoptosis and depletion of antioxidant defenses, consistent with a redox-mediated contribution [34,37].

It is useful at this point to compare experimental and human exposures directly, since the two are often discussed in the same paragraph without a stated relation. Taking the highest reported fasting peak plasma concentration of unconjugated EGCG in the purified-EGCG crossover study, 824.2 ng/mL after 500 mg [25], and the molar mass of EGCG (458.4 g/mol), the corresponding molar concentration is approximately 1.8 µmol/L. This is roughly five-fold below the lowest concentration at which injury was observed in primary rat hepatocytes and approximately 17- to 55-fold below the concentrations that impaired mitochondrial membrane potential in the same model [7]. The comparison has two implications that point in opposite directions, and both should be stated. It argues against peripheral plasma concentrations of unconjugated EGCG being sufficient, at ordinary exposures, to produce the direct cytotoxicity observed in vitro. At the same time, because absorbed EGCG reaches the liver through the portal circulation, hepatic concentrations may be appreciably higher than peripheral plasma concentrations, and this margin is therefore an upper bound on safety rather than a measurement of hepatic exposure [1]. The conversion above is an approximate arithmetic comparison of concentrations measured in different systems, not a validated in vitro-to-in vivo extrapolation.

Three further considerations qualify that arithmetic, and each works against treating the margin as a safety factor. First, “unconjugated” and “unbound” are not interchangeable. The human pharmacokinetic studies quantify unconjugated EGCG, meaning the parent compound rather than its glucuronide and sulfate conjugates; they do not report the protein-unbound fraction. EGCG binds plasma proteins, so the pharmacologically available concentration in vivo is lower than the measured unconjugated concentration, whereas the nominal concentrations used in hepatocyte experiments are added to media whose protein content is typically much lower than that of plasma. The two figures therefore overstate the comparability of the free concentrations on each side. Second, the exposure durations differ: the in vitro injury thresholds cited above follow 24 h of continuous exposure [7], whereas human plasma concentrations after a single dose peak within roughly 1.5 to 2 h and decline with a half-life of about 2 h [6,15,25], so an equivalent peak concentration would not represent an equivalent exposure. Third, extracellular auto-oxidation of EGCG in bicarbonate-buffered media generates hydrogen peroxide independently of cells [32], so part of the in vitro effect at a given nominal concentration may not be reproduced at the same concentration in vivo.

Because hepatic tissue concentrations of EGCG have not been measured in humans, these considerations cannot be resolved into a quantitative estimate of hepatic exposure, and no human toxicity threshold is derived here from the animal doses or in vitro concentrations discussed above. The comparison is offered to make explicit a relation that is usually left implicit, and its direction—that peripheral plasma concentrations of unconjugated EGCG sit below in vitro injury thresholds while hepatic concentrations remain unknown—is the most that the available data support [1].

Nevertheless, mechanisms demonstrated in experimental models should not be regarded as the sole established mechanism of green tea extract-associated liver injury in humans [5]. The strong association between clinical liver injury and HLA-B*35:01 [9], together with experimentally demonstrated T-cell responses to EGCG [39], suggests a role for individual and immunogenetic susceptibility in human toxicity.

### 7.4. Immune-Mediated and Idiosyncratic Mechanisms

The clinical phenotype of green tea-associated liver injury differs from that expected of a purely concentration-dependent hepatotoxin. Injury occurs in a small minority of users, across a wide range of nominal intakes, after variable latency, and with a hepatocellular pattern and frequent jaundice [5,9]. These features define idiosyncratic drug-induced liver injury, in which host response rather than dose alone determines outcome [40,41].

One current model of idiosyncratic injury holds that mitochondrial dysfunction, oxidative stress, and altered bile acid homeostasis place hepatocytes under stress and that in some individuals these stressed cells trigger an immune response that produces clinically significant injury [42]. This model connects the mechanisms described in Section 7.1 and Section 7.2 to the immunogenetic association rather than treating them as competing explanations. EGCG chemistry offers a plausible starting point for the immune step, since its autoxidation covalently modifies proteins [31]. Consistent with this, EGCG primed peripheral blood mononuclear cells from healthy donors—in 2 of 4 donors carrying HLA-B*35:01 and 1 of 4 not carrying it—and EGCG-specific T-cell clones generated from two allele-positive donors proliferated and secreted Th1 and Th2 cytokines together with granzyme B [39]. The limits of that experiment should be stated alongside its result. It was performed in cells from healthy volunteers rather than from patients with green tea-associated liver injury, so it demonstrates that EGCG can elicit a T-cell response, not that such a response caused injury in any patient. More directly, the allele restriction was tested by antibody blocking: clone proliferation was inhibited by MHC class II blocking antibodies but largely unaffected by HLA class I (HLA-A, -B and -C) blockade, and the authors concluded that the responses were driven primarily through MHC class II (HLA-DR) interactions and did not require expression of the class I risk allele. Consistent with that conclusion, priming was obtained from donors both carrying and not carrying HLA-B*35:01, and the responding clones were CD4-positive [39]. The experimental data therefore support an immune component in the response to EGCG, but they do not demonstrate an HLA-B*35:01-restricted mechanism, and the genetic association and the cellular findings should be read as two separate lines of evidence pointing to immune involvement rather than as one mechanism shown in two ways.

The clinical association is strong: HLA-B*35:01 was carried by 26 of 36 genotyped green tea-associated cases in the U.S. Drug-Induced Liver Injury Network (72%, 95% CI 58–87%), against 15% of patients with liver injury attributed to other dietary supplements, 12% of patients with conventional drug-induced liver injury, and 11% of population controls drawn from an imputed reference set of 15,094 individuals; the allele frequency in green tea cases was 0.417 against 0.057 in those population controls, approximately seven-fold higher [9]. The public health significance of the marker follows from that control frequency rather than from the case frequency: an allele carried by roughly one person in nine in the general population cannot, on its own, identify the small minority of extract users who develop liver injury. Its predictive value for an individual user is nevertheless low, and this distinction matters for practice. Carriage in roughly one in nine of the control population, set against the rarity of clinically apparent green tea-associated liver injury among exposed users, necessarily yields a low positive predictive value, so most carriers who take green tea extract will not develop liver injury [5,9]. Prospective genotype-based screening of asymptomatic users has not been evaluated, and the available data do not support it [9]. Oxidative stress and immune mechanisms are not competing explanations: reactive intermediates generated under conditions of high exposure provide the chemical starting point, while host immunogenetics determine whether that chemistry results in clinically significant injury [42,43].

## 8. Evidence of Hepatotoxicity

### 8.1. Cellular and Animal Studies

Experimental evidence supports the hepatotoxic potential of high EGCG exposure. In primary rat hepatocytes, 24-h exposure to EGCG concentrations of 10 µmol/L or higher caused cellular injury and functional loss; ROS responses were biphasic, and mitochondrial changes became more pronounced at higher concentrations [7]. In mice, repeated intragastric bolus dosing with 500–750 mg/kg EGCG for three days produced hepatic inflammation, necrosis, and haemorrhage together with markers of oxidative stress and depletion of antioxidant enzymes [8]. Dose, route, and dosing pattern are consistent determinants of severity across these models: bolus gavage produces greater injury than the same daily intake given in the diet [37], and repeated dosing with pure EGCG in rats shows dose- and route-dependent hepatotoxicity [27].

The host nutritional state modifies this response. In female C57BL/6J mice, intragastric EGCG at 400 or 800 mg/kg produced no significant change in plasma ALT, AST, or total bile acids and no histological injury in freely fed animals but caused dose-dependent elevations in all three measures, vacuolar degeneration with inflammatory infiltration, and increased hepatocyte apoptosis in animals that had received 50% of normal food intake for six days [26]. Plasma metabolomics in the same study linked the combined condition to accumulation of pro-inflammatory linoleic and arachidonic acid oxidation products and to disruption of phosphatidylcholine and sphingomyelin–ceramide turnover. The accompanying rise in total bile acids is notable because it points to disturbance of hepatobiliary transport alongside hepatocellular injury [26]; disturbed bile acid homeostasis is one of the mechanisms proposed to underlie hepatocyte injury in drug-induced liver injury generally [42]. These findings identify dietary restriction as an experimentally demonstrated modifier of EGCG hepatotoxicity, but the exposure condition is sustained caloric restriction rather than the overnight fast used in the human pharmacokinetic studies discussed in Section 6 [26].

The distance between that model and human weight-loss practice deserves an explicit statement, because the animal finding is the single most suggestive piece of evidence connecting nutritional state to hepatic risk and is correspondingly easy to over-read. Four differences are material. The intervention was a fixed 50% reduction in intake maintained continuously for six days in inbred mice, whereas human weight-loss patterns are heterogeneous in depth, duration and composition, ranging from modest sustained deficits to intermittent fasting regimens with normal total intake. The EGCG doses, 400 and 800 mg/kg by gavage, exceed human supplemental intake by two to three orders of magnitude on a body-weight basis and were delivered as a single bolus. Rodents have a higher mass-specific metabolic rate and deplete hepatic glycogen far more rapidly than humans, so six days of restriction in a mouse is not metabolically equivalent to six days of dieting in a person. And the readouts were obtained in an inbred strain, which removes the genetic heterogeneity that appears to govern the human phenotype [26].

The appropriate inference is therefore narrow. The experiment establishes that host nutritional state can convert a non-hepatotoxic EGCG dose into a hepatotoxic one in a mammalian liver, which is proof of the principle that the variable matters. It does not quantify risk in people who restrict energy intake, and it should not be used to estimate hazard for individuals using green tea extract during weight loss. That combination has not been studied prospectively in humans, and the caution expressed in Section 10 rests on precaution and biological plausibility rather than on demonstrated human risk [5,26].

Although these findings demonstrate that high EGCG exposure can produce biologically meaningful toxicity, the experimental doses and conditions are not equivalent to usual human consumption [1]. Micromolar cell-culture concentrations and high mg/kg animal doses cannot be directly equated with human plasma or hepatic tissue exposure, and the approximate margin between them is quantified in Section 7.3. In vitro results additionally require the medium-dependent auto-oxidation caveat discussed in Section 7.1 [32]. These results should therefore be interpreted as mechanistic and biological-plausibility evidence rather than direct proof of human hepatotoxicity [5].

### 8.2. Controlled Human Studies

In controlled human data on high-dose green tea extract, postmenopausal women who received decaffeinated extract providing approximately 843 mg EGCG/day for one year developed ALT elevations at rates of 6.7% in the extract group and 0.7% in the placebo group; 1.3% of extract recipients experienced an ALT-related serious adverse event [44]. The administration conditions of that trial are relevant to the present question and are therefore stated here: participants were instructed to take two capsules at breakfast and two capsules at dinner daily for 12 months, each capsule providing approximately 210.7 mg EGCG, giving a mean intake of 843 mg EGCG within 1315 mg total catechins per day [44]. The prescribed regimen was thus a divided dose taken with meals, not a fasting bolus. In the hepatic safety analysis of the same Minnesota Green Tea Trial among participants with normal baseline liver enzymes, increases in ALT and AST were greater with extract than placebo, and 26 of 513 extract users (5.1%) developed moderate or more severe liver biochemical abnormalities, with enzyme decline and recurrence during dechallenge and rechallenge [10].

Prescribed administration conditions should be distinguished from verified adherence. Compliance in that trial was monitored, but the timing of each dose relative to food intake was not analytically verified for individual participants, so the trial documents what participants were instructed to do rather than what each of them did [10,44]. This distinction bounds the inference in both directions. The occurrence of aminotransferase elevations, including serious events, under a regimen prescribed with meals shows that taking a concentrated extract with food does not eliminate hepatic risk. It does not, however, establish the comparative risk of fasting administration, because the trial contained no fasting arm; the comparison that would settle the question has not been performed in humans.

These findings are consistent with the broader intervention literature. A systematic review of adverse-event data from 159 human intervention studies found that hepatic adverse events occurred in a dose-dependent manner with a limited range of concentrated, catechin-rich green tea preparations ingested as large solid bolus doses, but not when green tea was consumed as a brewed beverage or as part of food. On this basis the review derived a safe intake level of 338 mg EGCG/day for solid bolus preparations and an observed safe level of 704 mg EGCG/day for beverage forms [12]. These values are specific to the methodology and evidence base of that review, in which adverse-event reports from heterogeneous intervention studies were aggregated and stratified by product form, and they should be cited as such rather than as independently validated thresholds. Two limits on their interpretation follow. Aggregated comparisons across studies that differ in population, dose, duration, product and monitoring intensity can demonstrate that adverse events cluster with particular exposure patterns, but they cannot isolate the product matrix as the cause of that clustering, since dose, dosing pattern and matrix covary across the contributing studies. And a level derived from the absence of reported events in aggregate data describes the exposures at which events were not detected in those studies; it is not a guarantee of safety for an individual, particularly where the clinical phenotype is idiosyncratic and occurs across a wide exposure range [5,9]. A separate systematic review restricted to randomized controlled trials reached a compatible conclusion: across 34 trials, liver-related adverse events were reported in only four, consisted mostly of mild liver enzyme elevations, and included no serious events, with a pooled odds ratio of 2.1 whose confidence interval spanned no effect [13]. That result is informative about the frequency of detectable hepatic effects in trial populations rather than about rare idiosyncratic injury, which randomized trials are not designed to capture. An independent safety assessment likewise distinguished beverage from dried extract exposures when deriving acceptable intakes [14]. The absence of similar effects in all participants receiving the same nominal dose nevertheless indicates substantial interindividual variability [1,12]. The USP review likewise concluded that safety cannot be explained by daily EGCG dose alone because clinical liver injury has been reported across a wide exposure range and susceptibility varies between individuals [5].

A subsequent genetic secondary analysis of the Minnesota Green Tea Trial found greater ALT increases at months 6 and 9 among extract users with the UGT1A4 rs6755571 A/C genotype than among those with the C/C genotype. However, the analysis was limited to postmenopausal women, and 98% of the extract group was White [45]. A glucuronidation-related variant is mechanistically plausible given the role of UDP-glucuronosyltransferases in catechin conjugation [21], but a secondary analysis in a single demographically narrow cohort cannot establish it. The strong association of HLA-B*35:01 with clinically apparent green tea-associated liver injury also supports genetic susceptibility, but this association was demonstrated in DILIN cases and does not establish the performance of prospective individual-risk prediction among asymptomatic extract users [9].

### 8.3. Clinical Liver Injury and DILIN Data

Among 1414 cases subjected to formal causality assessment by the U.S. Drug-Induced Liver Injury Network, 40 liver-injury episodes attributed to green tea occurred in 38 patients. Of these episodes, 95% showed a hepatocellular pattern, 83% involved jaundice, three required liver transplantation, and HLA-B*35:01 carriage was present in 72% of genotyped green tea cases [9]. Green tea products feature within the broader pattern of herbal and dietary supplement hepatotoxicity reported by the same network, in which such products accounted for 130 of 839 cases enrolled between 2004 and 2013 and rose from 7% to 20% of enrolled injuries over that period [46].

Asymptomatic aminotransferase elevation must be distinguished from clinical DILI. Aminotransferase increases may signal hepatocellular stress or injury but do not alone indicate symptomatic DILI, hepatocellular injury with jaundice, or acute liver failure. Clinical assessment should jointly consider ALT, alkaline phosphatase, and bilirubin concentrations; the R ratio; symptoms; exclusion of alternative causes; dechallenge/rechallenge patterns; and causality assessment [47,48]. Structured causality instruments, including RUCAM, are used for this purpose [49]; their application to herbal products is complicated by incomplete product and exposure characterization [50]. Serum aminotransferases and bilirubin remain the practical basis of assessment; mechanistic biomarkers of oxidative injury have been proposed but are not established for clinical decision-making [43].

The presence of numerous ingredients in herbal and dietary supplements can make attribution of causality to a single component difficult [50,51]. Product labels may not accurately reflect actual composition, and mislabelling, adulteration, and contamination have all been documented; concomitant exposures further complicate causality assessment [47,50].

Product-related uncertainty also complicates separation of EGCG-specific toxicity from risk attributable to the preparation. Safety assessment should consider labeled and analytically verified EGCG content, total catechin and caffeine content, extraction method, delivery systems, co-ingredients capable of modifying bioavailability, and pesticide, heavy-metal, or solvent residues. Concomitant hepatotoxic medications and other herbal supplements should also be investigated. Safety findings from purified EGCG, standardized single-ingredient extracts, and multi-ingredient weight-loss products should not be transferred automatically between product categories [5,47].

In most DILIN cases attributed to green tea, green tea was one component of a multi-ingredient preparation, and most products were administered as extract-containing tablets or capsules [9]. The proportions are substantial enough to bear on causality: of the 40 episodes, 8 (20%) involved a single-ingredient green tea product and 32 (80%) a multi-ingredient one containing between 4 and 60 declared active ingredients (median 11.5), with only 7 of those products naming green tea in the product title; green tea extract was present in tablet or capsule form in all but two products, and a green tea extract dose per serving was declared for only 17 of the 40 [9]. The USP review reported hepatotoxicity cases across an approximate EGCG exposure range of 140–1000 mg/day, substantial interindividual susceptibility, and no definable dose-based toxicity threshold. These data support that injury cannot be explained solely by total ingested EGCG [5]. An absent dose threshold is what an idiosyncratic, host-dependent mechanism predicts, and it is therefore consistent with the immunogenetic findings discussed in Section 7.4 rather than anomalous [40].

The confounding weight this carries should not be understated. Where a product declares a median of about a dozen active ingredients, attribution to EGCG rests on the aggregate pattern across cases—the consistency of the hepatocellular phenotype, the dechallenge and rechallenge observations, and above all the HLA-B*35:01 association, which is specific to the green tea cases rather than to supplement use in general [9,40]—rather than on component-level causality in any individual case. Several of the co-ingredients typical of weight-loss and sports preparations have themselves been implicated in liver injury; label composition is not reliably verified, and adulteration and mislabelling are documented in this product class [47,50,51]. For individual multi-ingredient cases, therefore, formal causality assessment can implicate the product but rarely the constituent, and estimates of green tea-attributable risk derived from such series carry an upward bias of unknown size.

Case series and DILI registries are valuable for characterizing clinical phenotype and potential susceptibility factors, but because they do not include the total exposed population, they cannot directly estimate the absolute incidence of hepatotoxicity in the community [51]. Controlled trials, clinical liver-injury registries, and regulatory safety assessments should therefore be regarded as complementary sources that answer different evidentiary questions [1]. Key experimental and human studies on the hepatic safety of EGCG and green tea extract are summarized in Table 3.

## 9. Evidence Chain Linking Fasting EGCG Exposure and Hepatotoxicity

When the available evidence is considered together, the first step of the chain is demonstrated directly by human pharmacokinetic data: administration of a standardized green tea catechin preparation under fasting conditions increases plasma Cmax and AUC for unconjugated EGCG [6]. The second step rests on experimental evidence: high EGCG concentrations are associated with increased ROS, loss of mitochondrial membrane potential, impaired oxidative phosphorylation, and hepatocellular injury, while high oral bolus exposure can cause oxidative stress, mitochondrial depletion, and hepatic injury in mice [7,8,37]. Animal evidence also links nutritional state to susceptibility, in that caloric restriction converts otherwise non-hepatotoxic EGCG doses into hepatotoxic ones in mice [26]. At the third step, controlled human data show that high-dose, prolonged green tea extract use can increase serum aminotransferases in some individuals [10], and aggregated human intervention data show that such events cluster among concentrated bolus preparations rather than beverage forms [12].

The transitions between these steps, rather than the steps themselves, are where the evidence is weakest. The first transition requires that increased systemic exposure translates into increased hepatic exposure, which has not been measured in humans [1]. The second requires that hepatic concentrations reach the range at which experimental injury occurs; the approximate margin between reported human peak plasma concentrations and in vitro injury thresholds is at least five-fold and plausibly larger (Section 7.3), and portal exposure is unquantified [7,25]. The third requires that concentration-dependent cytotoxicity accounts for clinical injury, yet the clinical phenotype is idiosyncratic and strongly associated with HLA-B*35:01, which indicates that host response is a necessary part of the explanation [9,40]. The chain is therefore not a single mechanism weakened by missing data, but a sequence in which at least one step probably operates by a different logic than the others.

No human study integrates these evidentiary steps into a single causal chain [1,5]. EFSA identifies fasting and bolus dosing as pharmacokinetic conditions that increase EGCG exposure but provides no controlled human data directly comparing clinical hepatotoxicity outcomes after the same EGCG dose under fasting and fed conditions [1]. The USP review acknowledges increased EGCG bioavailability during fasting and supports precautionary labeling against administration on an empty stomach, but it does not establish a clinical causal relationship showing that fasting alone causes hepatotoxicity in humans [5].

This distinction is central to clinical interpretation. Fasting should be considered a pharmacokinetic condition that can increase systemic exposure from concentrated EGCG preparations, rather than an independent, established hepatotoxic factor in humans. Because the direct evidence derives from standardized extract or catechin preparations, the magnitude of the effect should not be extrapolated to traditionally brewed green tea or to matcha [1,6].

**Figure 1:**
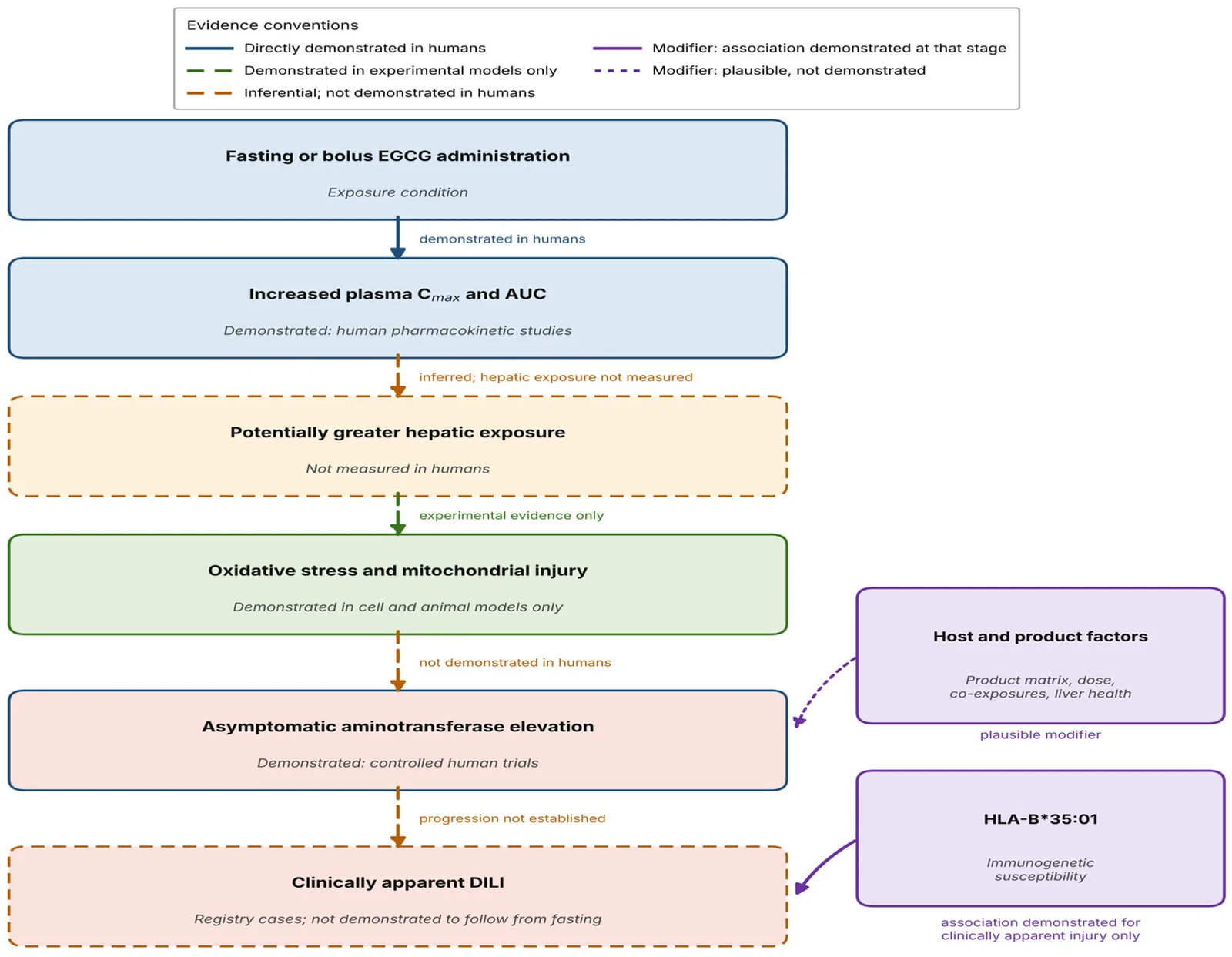
Evidence chain linking fasting systemic EGCG exposure and hepatotoxicity, with the strength of evidence for each transition shown explicitly. Line conventions are defined in the legend: a solid arrow marks a transition directly demonstrated in humans; a dashed arrow marks a transition demonstrated only in experimental models; a dashed arrow in the inferential colour marks a relationship that is biologically plausible but has not been demonstrated in humans. Boxes drawn with a dashed outline denote stages that have not been measured or established in humans. The green box with a solid outline marks a stage that has been demonstrated, but only in cellular and animal models; its colour corresponds to the green dashed arrow used for experimental evidence. Asymptomatic aminotransferase elevation and clinically apparent drug-induced liver injury are shown as separate stages because the first is documented in controlled trials, whereas progression from the first to the second has not been established. Modifiers are shown to the right and are not steps in the chain; the two modifier conventions are distinguished. HLA-B*35:01 is joined by a solid arrow to clinically apparent injury only, that being the stage at which the association has been demonstrated in registry cases subjected to formal causality assessment; no association with asymptomatic aminotransferase elevation has been established and none is implied. Host and product factors are joined to the laboratory-abnormality stage by a dotted arrow denoting a plausible but not demonstrated influence. The figure summarises the structure of the evidence and does not imply quantitative effect sizes.

Although the stages shown in Figure 1 form a biologically coherent evidence chain, the complete pathway from fasting to clinical DILI has not been demonstrated directly within a single human study.

## 10. Safety Assessment, Regulatory Approaches, and Clinical Recommendations

Safety assessment of green tea products should distinguish traditional infusions from concentrated extracts. EFSA considered traditionally prepared infusions generally safe at reported intake levels but found that EGCG intake of 800 mg/day or more as a food supplement was associated with increased serum transaminases in controlled intervention trials; it could not establish a definitive safe EGCG dose applicable to all individuals using green tea extracts [1]. Marked interindividual variability in clinical liver injury and the association with HLA-B*35:01 further indicate that a nominal daily dose alone is insufficient for safety assessment [9]. An independent systematic review of the same intervention literature approached the question differently, deriving separate intake levels for solid bolus preparations and for beverage forms rather than a single dose limit [12]. The relationship between those values and the EFSA position should be stated precisely, because they answer different questions. EFSA assessed whether a safe supplemental EGCG dose could be established for all users and concluded that it could not, given idiosyncratic injury and interindividual variability [1]. The systematic review asked at which aggregate exposures adverse events had and had not been reported in the published intervention literature and reported levels on that basis [12]. The second does not supersede or resolve the first: an exposure at which events were not detected across heterogeneous studies is not equivalent to a dose established as safe for an individual, and the values are not regulatory limits. The regulatory figures discussed below are different again, being risk-management decisions that incorporate exposure assessment, product category and labelling feasibility alongside the toxicological evidence. What the assessments do share, across methods and scope [5,11,13,14], is the finding that product form and conditions of use, and not nominal dose alone, shape hepatic risk.

Regulatory frameworks apply the same evidence base to different contexts of use. Commission Regulation (EU) 2022/2340 requires the daily portion of foods containing green tea extract to provide less than 800 mg EGCG and labels to state the maximum number of daily portions and warn against consuming 800 mg EGCG/day or more. It also requires warnings against consumption on the same day as other green tea products, use by pregnant or breastfeeding women and persons younger than 18 years, and consumption on an empty stomach [52]. This regulatory limit does not imply that every dose below 800 mg is risk-free for every individual.

The 2024 UK Committee on Toxicity assessment found no new evidence that invalidated the 2018 EFSA opinion but noted that idiosyncratic injury below 800 mg/day could not be excluded and that genetic characteristics, fed or fasting state, and general liver health may influence risk [53]. For defined green tea extracts used in supplemented foods, Health Canada adopted a recommended maximum supplemental intake of 300 mg EGCG/day and a maximum of 100 mg per serving, emphasizing that this exposure pattern differs from bolus capsules or fasting administration [54]. These differences demonstrate that dose values should not be interpreted independently of the product matrix and conditions of use.

The value of 800 mg EGCG/day should not be interpreted as a universal toxicity threshold or safe upper limit for all forms of green tea. EFSA estimated EGCG exposure from traditional infusions at up to 866 mg/day among high consumers yet considered traditional infusions generally safe at reported intake levels and explicitly noted that the product matrix may modify catechin pharmacokinetics and toxicity [1]. This apparent inconsistency is informative rather than contradictory, and the resolution lies in exposure pattern rather than in dose. Infusion intake is distributed across the day in a dilute aqueous matrix taken with or between meals, whereas supplement intake is typically a concentrated solid bolus that may be taken fasting; the same nominal daily amount therefore produces different peak systemic exposures [6,12,25]. Manufacturing, EGCG concentration, bolus dosing, and fasting can create a different systemic exposure pattern with concentrated extracts [5,12].

Increased systemic EGCG exposure under fasting conditions should be considered in the safety assessment of concentrated extracts. The warning in the USP Powdered Decaffeinated Green Tea Extract monograph advises that compliant products not be taken on an empty stomach and be consumed with food. It also advises individuals with liver disorders not to use the product and users who develop abdominal pain, dark urine, or jaundice to discontinue use and consult a healthcare professional [5]. EFSA recommends that green tea products, particularly supplements, disclose catechin content and the proportion of EGCG [1]. Because the catechin content of both infusions and extracts varies substantially with raw material, processing, and preparation [2,17], and because EGCG stability in formulation is limited [19], analytically verified rather than nominal EGCG content is the appropriate basis for such labeling.

Clinical evaluations should include a detailed history of herbal and dietary supplement use, including green tea extract. Product name and composition, duration and amount of use, multi-ingredient formulations, concomitant medications, and other supplements should be considered in causality assessment [5,47,51]. Where green tea extract is suspected, assessment follows the general approach to suspected drug- and supplement-induced liver injury, including exclusion of competing causes and structured causality evaluation [48,49]. Although HLA-B*35:01 is strongly associated with green tea-related DILI, available studies have not evaluated its prospective performance for predicting individual hepatotoxicity risk among asymptomatic extract users [9].

Genetic and metabolic susceptibility data should not yet be interpreted as definitive tools for individual risk prediction. The association between UGT1A4 rs6755571 and greater transaminase changes derives from a secondary analysis of the same Minnesota Green Tea Trial in postmenopausal and predominantly White participants [45]. Current evidence is insufficient to support routine clinical screening based solely on HLA-B*35:01 or UGT1A4 genotype, and the low positive predictive value expected for a common allele and a rare outcome is the principal reason (Section 7.4). Product characteristics, EGCG content, conditions of use, individual clinical factors, and emerging hepatic findings should be evaluated together [5,9].

Where a concentrated extract is used during intentional weight loss, the combination of restricted intake and high-dose EGCG deserves particular caution. Direct human data on this combination are not available, and the supporting evidence is currently limited to an animal model [26], but the precautionary advice already issued against administration on an empty stomach [5,52] applies with at least equal force in this setting. This is also the setting in which the three nutritional states distinguished in Section 5 converge, since weight-loss use may combine reduced energy intake with bolus administration and fasting.

### Practical Points for Clinicians and Dietitians

The evidence reviewed here supports a small number of operational points for practice. In taking a history, supplement use should be asked about directly and by category rather than by the open question of whether the patient takes any medication, since green tea extract is frequently consumed within weight-loss, sports or “detox” preparations whose names do not mention green tea [9,51]. Where use is reported, the useful details are the product name and manufacturer, the declared EGCG or total catechin content per serving, the number of servings per day, whether doses are taken with food or on an empty stomach, the start date and any interruption and resumption, concurrent medicines and other supplements, and alcohol intake; asking the patient to bring the container or photograph the label is more reliable than recall of the product name alone [5,47].

In counselling, the practical distinctions are between product forms rather than between doses alone: brewed green tea and matcha at customary intakes are not the exposure of concern, whereas concentrated extracts taken as capsules or tablets are, particularly at or above 800 mg EGCG/day, when taken on an empty stomach, or when combined with other catechin-containing products [1,52]. Advising that concentrated extracts be taken with food is a reasonable precaution that follows the pharmacokinetic evidence, provided it is presented as risk reduction rather than as elimination of risk, since aminotransferase elevations occurred in a trial whose regimen was prescribed with meals [10,44]. Particular caution is warranted where a concentrated extract is used during intentional energy restriction, a combination not studied prospectively in humans [26].

Where liver injury is suspected, the extract should be stopped and liver biochemistry interpreted using ALT, alkaline phosphatase and bilirubin together with the R ratio rather than aminotransferases alone, with competing causes excluded and a structured causality instrument applied [47,48,49]. Patients should be advised not to resume the product, as recurrence on re-exposure with shortened latency has been documented [9]. Genotyping for HLA-B*35:01 has no established role in this assessment and should not be offered as a screening test to asymptomatic users for the reasons set out in Section 7.4 [9].

## 11. Conclusions and Research Needs

The central conclusion of this review concerns the structure of the evidence rather than a new safety threshold. Fasting is an exposure-modifying pharmacokinetic condition, demonstrated to raise systemic EGCG exposure from concentrated preparations [6,25]; the mechanistic capacity of high EGCG exposure to injure hepatocytes is established in experimental systems [7,8,37]; and clinically apparent injury in humans is idiosyncratic and immunogenetically conditioned [9,39]. These three findings are frequently presented as successive stages of one causal process. They are better understood as evidence of different kinds, and the transitions between them, rather than the findings themselves, are what remain unestablished (Table 4).

Not all forms grouped under the term “green tea” are equivalent in hepatic safety. Traditionally prepared infusions are generally considered safe at reported intake levels, with rare idiosyncratic injury reported [1]; with concentrated extracts, aminotransferase elevations occur in controlled trials and cluster among solid bolus preparations rather than beverage forms [1,12]. The association of clinically apparent injury with HLA-B*35:01 indicates that this outcome is not a simple dose-dependent process [9,39].

Current human data do not establish that fasting alone causes clinical hepatotoxicity or increases its incidence [1,5,6]. Warnings against taking concentrated green tea extracts on an empty stomach should therefore be viewed as precautionary risk-reduction measures and should not be extrapolated directly to traditional infusions or matcha [1]. Framing the question this way identifies which precautions follow from demonstrated exposure effects and which would require evidence that does not yet exist.

Four research priorities follow directly from the gaps identified here. First, human studies should directly compare the same EGCG dose under fasting and fed conditions with adequate sample sizes and prospective hepatic safety outcomes. The two available randomized fed-versus-fasting comparisons were designed as pharmacokinetic studies in 30 and 10 participants respectively; neither included a hepatic endpoint, and the more recent did not report a significance level for the direct fasting-versus-fed contrast [6,15]; the gap is therefore one of design and power rather than of age. A trial able to answer the clinical question would need, as minimum design elements: a randomized crossover or parallel allocation of the same characterised EGCG dose to fasting and fed administration; a defined and analytically verified product, with separate arms or strata for purified EGCG, standardised single-ingredient extract and beverage matrix, since product form is itself a determinant of risk; serial plasma EGCG measurement sufficient to establish Cmax and AUC under each condition; hepatic safety as a prespecified outcome, with ALT, AST, alkaline phosphatase and bilirubin measured on a schedule able to detect the delayed elevations seen in long-term trials, together with prespecified stopping and dechallenge rules; stratification or prespecified subgroup analysis by HLA-B*35:01 and by relevant metabolic genotypes; duration sufficient for the latency observed in registry cases rather than single-dose administration; and a sample size derived from the incidence of aminotransferase elevation observed in high-dose extract trials rather than from pharmacokinetic variance [9,10,45]. Second, given the animal finding that caloric restriction converts non-hepatotoxic EGCG doses into hepatotoxic ones [26], studies in people using green tea extract during intentional energy restriction are a specific priority. Third, comparative studies of actual systemic EGCG exposure from traditional infusions, matcha, and different extract formulations are needed, together with any measurement that constrains the relationship between plasma and hepatic concentrations [1]. Fourth, the predictive performance of HLA-B*35:01 and metabolic variants should be evaluated prospectively in exposed but asymptomatic users before any screening role is considered [9,45]. Clarifying hepatic tissue exposure and the effects of product matrix and genetic susceptibility will help identify the product forms and individual characteristics associated with greater clinical risk [1,45].

## Figures and Tables

**Table 1:** Eligibility criteria applied in selecting evidence for this review.

Evidence Domain	Included	Excluded
Human pharmacokinetics	Studies administering a characterised green tea catechin preparation or purified EGCG and reporting plasma EGCG under defined fed or fasting conditions	Studies without a stated fed or fasting condition, or without quantified plasma catechin measurement
Controlled human safety	Randomized or controlled intervention studies reporting liver biochemistry with a defined EGCG dose and product	Uncontrolled series; studies in which EGCG exposure could not be separated from other interventions
Clinical liver injury	Registry or cohort reports applying a formal causality assessment, with product and exposure characterisation	Single case reports without causality assessment; reports in which the product was not identified
Experimental toxicology	Cell and animal studies reporting EGCG concentration or dose, route, and duration, with a defined hepatic endpoint	Studies of green tea fractions or mixtures in which the EGCG contribution could not be isolated
Regulatory assessment	Full-text opinions, monographs and statements issued by a national or supranational authority	Secondary summaries of regulatory positions; drafts superseded at the time of consultation
Conclusion-bearing use	Primary sources read in full	Findings available only through secondary citation; these were used for context but not to support principal conclusions

**Table 2:** Human studies directly examining the effects of fasting or fed status on EGCG exposure.

Source	Design	Product and Dose	Fasting and Comparator	Pharmacokinetic Result	Hepatic Outcome	Key Limitation
Chow et al. [6]	Randomized crossover; 30 healthy participants, *n* = 10 per dose	Polyphenon E; 400, 800, or 1200 mg EGCG	No food after overnight fast versus light breakfast	Unconjugated EGCG Cmax > 3.5-fold higher; AUC higher at all doses	Not assessed	Small dose groups; no tissue-exposure or clinical DILI outcome; the fed-versus-fasting contrast has since been re-examined in a second small crossover study [15]
Andreu-Fernández et al. [15]	Randomized three-condition crossover; *n* = 10 (5 men, 5 women)	Teavigo, 300 mg extract standardized to 94% EGCG; FontUp ≥ 250 mg EGCG	≥8-h overnight fast; Teavigo without breakfast versus Teavigo with a 480-kcal breakfast; FontUp in milk	Cmax fasting versus fed (men/women) 528.6/466.1 versus 275.3/318.7 ng/mL; AUC0–360 also higher under fasting; significant differences were reported for comparisons with FontUp (AUC0–360 *p* = 0.036 in men, *p* = 0.001 in women)	Not assessed	No *p* value reported for the direct fasting-versus-fed contrast of Cmax or AUC; stated EGCG dose internally inconsistent; small sample; 6-h sampling; sex-stratified reporting
Naumovski et al. [25]	Three-condition crossover; *n* = 4	500 mg purified EGCG	≥10 h fasting; capsule with water, capsule with breakfast, or sorbet	Fasting-capsule AUC 2.7- and 3.9-fold higher; Cmax 824.2 versus 231.8 and 218.0 ng/mL	Not assessed	Very small sample; only 8-h pharmacokinetic follow-up

**Table 3:** Key experimental and human studies on the hepatic safety of EGCG and green tea extract.

Source	Design/Sample	EGCG Exposure	Main Finding	Key Interpretive Limitation
Chow et al. [6]	Randomized crossover pharmacokinetic study; 30 healthy participants, *n* = 10 per dose group	Single-dose Polyphenon E: 400, 800, or 1200 mg EGCG; overnight fast versus light breakfast	Fasting increased unconjugated EGCG Cmax by >3.5-fold; AUC significantly higher at all three doses	No liver-injury outcome or hepatic tissue EGCG measurement; only 10 participants per dose group; the fed-versus-fasting contrast has since been re-examined in a second small crossover study [15]
Andreu-Fernández et al. [15]	Randomized three-condition crossover pharmacokinetic study; *n* = 10 healthy volunteers (5 men, 5 women)	Teavigo, 300 mg extract standardized to 94% EGCG, fasting or with a 480-kcal breakfast; FontUp (≥250 mg EGCG) in milk; ≥8-h overnight fast before each condition	Mean Cmax higher under fasting than with breakfast (men 528.6 versus 275.3 ng/mL; women 466.1 versus 318.7 ng/mL); AUC0–360 also numerically higher under fasting	No significance level reported for the direct fasting-versus-fed contrast; stated EGCG content of the test product internally inconsistent; 6-h sampling; no hepatic exposure or liver-injury outcome
Naumovski et al. [25]	Three-condition crossover pharmacokinetic study; 4 healthy participants	500 mg purified EGCG after ≥10 h fasting: capsules with water, capsules with breakfast, or incorporated into sorbet	Fasting-capsule AUC 2.7-fold higher than with breakfast and 3.9-fold higher than with sorbet; Cmax 824.2, 231.8, and 218.0 ng/mL	Very small sample; hepatic safety and tissue exposure were not assessed
Kucera et al. [7]	In vitro; primary hepatocytes isolated from male Wistar rats, with mitochondrial respiration measured in permeabilized hepatocytes. The source states that experiments were performed at least three times; the number of animals, the number of independent hepatocyte isolations and the number of technical replicates per condition are not reported.	24-h EGCG exposure; signs of injury at ≥10 µmol/L	ROS decreased at low concentrations but increased at the highest; mitochondrial membrane potential fell at 30 and 100 µmol/L; high concentrations uncoupled oxidative phosphorylation and damaged the outer mitochondrial membrane	In vitro rat-hepatocyte model; concentrations are not directly equivalent to human oral exposure, and medium-dependent auto-oxidation may contribute
Lambert et al. [37]	Mouse model of oral EGCG hepatotoxicity	High oral EGCG doses	High oral doses of EGCG produced hepatotoxicity, establishing an oral dose range at which hepatic injury occurs in mice	High experimental doses in a mouse model; not directly generalizable to human intake
James et al. [8]	Male C57BL/6J mice	Intragastric EGCG 500–750 mg/kg once daily for 3 days	Hepatic inflammation, necrosis, and haemorrhage; increased oxidative stress with decreased superoxide dismutase and glutathione peroxidase; decreased hepatic mitochondrial copy number and sirtuin 3 expression	High experimental mg/kg doses and mouse model; not directly generalizable to usual human consumption
Ramachandran et al. [27]	Repeated-dose toxicity study in rats; oral and intraperitoneal routes	Pure EGCG, repeated dosing over 14 days at graded dose levels	Dose- and route-dependent hepatotoxicity with associated dyslipidemia	Rodent model with supraphysiological exposures; route dependence limits direct translation to oral human use
Shi et al. [26]	Female C57BL/6J mice; free diet versus 50% caloric restriction for 6 days; *n* = 8 per group	Single intragastric 400 or 800 mg/kg EGCG under each feeding condition	No liver injury with EGCG alone or restriction alone; combined condition produced dose-dependent ALT, AST, and total bile acid elevations, vacuolar degeneration, and hepatocyte apoptosis	Mouse model; exposure is sustained caloric restriction, not the overnight fast used in human pharmacokinetic studies
Dostal et al. [44]	Randomized double-blind placebo-controlled Minnesota Green Tea Trial; 1075 postmenopausal women	Decaffeinated GTE providing approximately 843 mg EGCG/day within 1315 mg total catechins for 12 months, taken as two capsules at breakfast and two capsules at dinner (approximately 210.7 mg EGCG per capsule)	ALT elevation in 6.7% with GTE versus 0.7% with placebo; ALT-related serious adverse event in 1.3% of the GTE group	Postmenopausal, predominantly White population; limited generalizability; regimen prescribed with meals, with individual timing relative to food not analytically verified
Yu et al. [10]	Hepatic safety analysis of the same trial; normal baseline enzymes, *n* = 1021 (513 GTE, 508 placebo)	Same intervention: approximately 843 mg EGCG/day for 12 months	ALT +5.4 U/L (95% CI 3.6–7.1) and AST +3.8 U/L (95% CI 2.5–5.1) with GTE versus −0.5 and −0.2 U/L with placebo (both *p* < 0.001); grade 2 or higher abnormality in any of ALT, AST, alkaline phosphatase or total bilirubin in 26/513 GTE participants (5.1%) versus 4/508 on placebo (0.8%), adjusted OR 7.0 (95% CI 2.4–20.3); dechallenge–rechallenge pattern	Not an independent cohort from Dostal et al.; hepatic safety analysis of the same RCT; the endpoint is a laboratory grade threshold (aminotransferases > 2.5× ULN; bilirubin > 1.5× ULN), not clinically apparent liver injury
Hu et al. [12]	Systematic review of toxicology studies and adverse-event data from 159 human intervention studies	Range of green tea, GTE, and EGCG interventions in beverage, food, and solid bolus forms	Hepatic adverse events dose-dependent and limited to concentrated, catechin-rich preparations taken as large solid bolus doses. On this basis the review derived a safe intake level of 338 mg EGCG/day for solid bolus preparations and an observed safe level of 704 mg EGCG/day for beverage forms. Both figures are values derived within that review from aggregated adverse-event data; they are neither validated individual safety thresholds nor regulatory limits	Aggregated adverse-event data of heterogeneous design; not a controlled comparison of fed versus fasting clinical outcomes. Dose, dosing pattern and product matrix covary across the contributing studies, so the derived levels describe exposures at which events were not detected rather than doses established as safe for an individual (see Section 8.2)
Isomura et al. [13]	Systematic review of 34 randomized controlled trials of green tea extracts	Green tea extract interventions across trial dose ranges	Liver-related adverse events were reported in 4 of 34 trials, consisted mostly of mild liver enzyme elevations, and included no serious events; the pooled odds ratio versus placebo was 2.1 with a confidence interval spanning no effect	Randomized trials exclude the rare idiosyncratic cases captured by registries; liver-outcome reporting was heterogeneous and the pooled estimate imprecise
Hoofnagle et al. [9]	U.S. DILIN; 40 episodes in 38 patients attributed to green tea among 1414 formally adjudicated cases	Clinical product use; mostly extract-containing tablets/capsules and multi-ingredient products	95% hepatocellular pattern, 83% jaundice, 3 transplantations; HLA-B*35:01 in 72% of genotyped cases versus 11% of population controls	Selected DILI cases do not indicate absolute population risk; many used multi-ingredient products
Line et al. [39]	In vitro; peripheral blood mononuclear cells from 4 HLA-B*35:01-positive and 4 HLA-B*35:01-negative healthy donors; T cells from additional non-HLA-typed healthy donors co-cultured with autologous monocyte-derived dendritic cells; EGCG-specific T-cell clones generated from 2 HLA-B*35:01-positive donors. The number of clones, the number of donors in the dendritic-cell priming experiment and the number of technical replicates per condition are not reported.	EGCG exposure in culture	EGCG primed peripheral blood mononuclear cells in 2 of 4 allele-positive and 1 of 4 allele-negative donors, with secretion of IFN-γ, IL-5, IL-13 and granzyme B; T-cell priming was enhanced by PD-1 and PD-L1 blockade. Clones were CD4-positive, and their proliferation was inhibited by MHC class II blocking antibodies but largely unaffected by HLA class I (HLA-A, -B, -C) blockade	Healthy-donor in vitro model; demonstrates immune reactivity to EGCG rather than clinical injury. The authors conclude that the responses were driven primarily through MHC class II (HLA-DR) and did not require expression of the class I risk allele, so the experiment does not establish an HLA-B*35:01-restricted mechanism; a contribution from CD8-positive T cells was not excluded
Acosta et al. [45]	Genetic secondary analysis of the Minnesota Green Tea Trial; GTE *n* = 400, rs6755571 *n* = 386	Approximately 843 mg EGCG/day for 12 months	ALT increases at months 6 and 9 greater with the UGT1A4 rs6755571 A/C genotype than C/C	Not an independent clinical study; same trial, postmenopausal women, and 98% of GTE group was White

**Table 4:** Summary of evidence levels for the relationship between fasting, systemic EGCG exposure, and hepatotoxicity.

Question	Established Human Evidence	Experimental Evidence	Unresolved Causal Question	Clinical Implication
Does fasting increase systemic EGCG exposure?	Yes: >3.5-fold higher unconjugated EGCG Cmax and higher AUC after an overnight fast in a randomized crossover study (*n* = 30), with the same direction of effect in a second randomized crossover (*n* = 10) and in a four-participant crossover with purified EGCG [6,15,25]	Consistent with capacity-limited presystemic metabolism and efflux, though the mechanism has not been isolated [20,21,24]	Whether the effect size generalizes beyond standardized catechin preparations to infusions and matcha, and the magnitude of the fed-versus-fasting difference, which the more recent crossover did not test formally	Take concentrated extracts with food; do not transfer the effect size to brewed tea [5,52]
Does increased systemic exposure raise hepatic exposure?	Not measured; human tissue distribution data are limited [1]	Portal delivery precedes systemic distribution, so hepatic concentrations may exceed peripheral plasma; the size of the difference in humans is not established [1]	The quantitative relationship between plasma and hepatic EGCG concentrations in humans	Plasma concentrations should not be used as a surrogate for hepatic exposure in risk assessment
Can high EGCG exposure injure hepatocytes?	No human study administers defined hepatic concentrations	Yes: injury from ≥10 µmol/L, mitochondrial membrane potential loss at 30–100 µmol/L in primary hepatocytes, and dose- and route-dependent injury in rodents [7,27,37]	Whether concentrations in this range are ever reached in human liver; reported human peak plasma concentrations are roughly 1.8 µmol/L (Section 7.3)	Mechanistic plausibility is established; it does not by itself quantify human risk
Does nutritional restriction modify toxicity?	No human data; the combination of energy restriction and high-dose extract has not been studied prospectively	Yes: 50% caloric restriction for six days converted non-hepatotoxic EGCG doses into hepatotoxic ones in mice [26]	Whether intentional energy restriction during weight loss alters hepatic risk in people	Particular caution where concentrated extracts are used during intentional weight loss
Does high-dose extract use raise liver enzymes in humans?	Yes: ALT elevation in 6.7% versus 0.7% with placebo, and moderate or worse abnormality in 5.1% of extract users, with dechallenge–rechallenge [10,44]	Consistent with experimental hepatotoxicity at high exposures [8,34]	Why only a minority of recipients of the same nominal dose are affected	Monitor symptomatic users; asymptomatic elevation is not equivalent to clinical DILI [47]
Is clinical injury explained by dose?	No: cases span approximately 140–1000 mg EGCG/day with no definable threshold, and 72% of genotyped DILIN cases carried HLA-B*35:01 [5,9]	EGCG-specific T-cell responses in HLA-B*35:01 carriers support an immune component [39,42]	Which additional host factors determine who progresses from exposure to injury	Dose limits reduce but do not eliminate risk; genotype screening is not currently supported [9]
Does fasting cause clinical hepatotoxicity?	Not established; no controlled comparison of clinical outcomes under fasting versus fed conditions exists [1,5]	Indirect support only, via exposure and via caloric restriction in mice [12,26]	Whether the same EGCG dose produces more hepatic injury when taken fasting in humans	Empty-stomach warnings are precautionary risk reduction, not established causal prevention [5,52]

## Data Availability

No new data were created or analyzed in this study. Data sharing is not applicable to this article.

## Abbreviations

The following abbreviations are used in this manuscript:

ALT	alanine aminotransferase
AST	aspartate aminotransferase
AUC	area under the concentration–time curve
BCRP	breast cancer resistance protein
Cmax	maximum plasma concentration
COMT	catechol-O-methyltransferase
COT	Committee on Toxicity of Chemicals in Food, Consumer Products and the Environment
DILI	drug-induced liver injury
DILIN	Drug-Induced Liver Injury Network
EC	epicatechin
ECG	epicatechin-3-gallate
EFSA	European Food Safety Authority
EGC	epigallocatechin
EGCG	epigallocatechin-3-gallate
GTE	green tea extract
HLA	human leukocyte antigen
Nrf2	nuclear factor erythroid 2-related factor 2
OR	odds ratio
PCR	polymerase chain reaction
RCT	randomized controlled trial
ROS	reactive oxygen species
RUCAM	Roussel Uclaf Causality Assessment Method
SULT	sulfotransferase
UGT1A4	uridine 5′-diphospho-glucuronosyltransferase 1A4
USP	United States Pharmacopeia
